# Renal impairment and use of nephrotoxic agents in patients with multiple myeloma in the clinical practice setting in the United States

**DOI:** 10.1002/cam4.1075

**Published:** 2017-06-14

**Authors:** Yi Qian, Debajyoti Bhowmik, Christopher Bond, Steven Wang, Sam Colman, Rohini K. Hernandez, Paul Cheng, Michele Intorcia

**Affiliations:** ^1^ Global Health Economics Amgen Inc. Thousand Oaks California; ^2^ Covance Gaithersburg Gaithersburg Maryland; ^3^ Center for Observational Research Amgen Inc. Thousand Oaks California; ^4^ Global Development Amgen Inc. Thousand Oaks California

**Keywords:** Chronic kidney disease, intravenous bisphosphonate, multiple myeloma, nephrotoxic agent, renal impairment

## Abstract

Renal impairment is a common complication of multiple myeloma and deterioration in renal function or renal failure may complicate clinical management. This retrospective study in patients with multiple myeloma using an electronic medical records database was designed to estimate the prevalence of renal impairment (single occurrence of estimated glomerular filtration rate [eGFR] <60 mL/min per 1.73 m^2^ on or after multiple myeloma diagnosis) and chronic kidney disease (at least two eGFR values <60 mL/min per 1.73 m^2^ after multiple myeloma diagnosis that had been measured at least 90 days apart), and to describe the use of nephrotoxic agents. Eligible patients had a first diagnosis of multiple myeloma (ICD‐9CM: 203.0x) between January 1, 2012 and March 31, 2015 with no prior diagnoses in the previous 6 months. Of 12,370 eligible patients, the prevalence of both renal impairment and chronic kidney disease during the follow‐up period was high (61% and 50%, respectively), and developed rapidly following the diagnosis of multiple myeloma (6‐month prevalence of 47% and 27%, respectively). Eighty percent of patients with renal impairment developed chronic kidney disease over the follow‐up period, demonstrating a continuing course of declining kidney function after multiple myeloma diagnosis. Approximately 40% of patients with renal impairment or chronic kidney disease received nephrotoxic agents, the majority of which were bisphosphonates. As renal dysfunction may impact the clinical management of multiple myeloma and is associated with poor prognosis, the preservation of renal function is critical, warranting non‐nephrotoxic alternatives where possible in managing this population.

## Introduction

Renal impairment is a common complication of multiple myeloma, with a complex pathophysiology and various underlying risk factors 1, 2. The toxicity of immunoglobulin light chain deposition in the renal tubules is the chief cause of renal dysfunction in these patients. A variety of factors may contribute to renal damage including hypercalcemia of malignancy, dehydration, infections, amyloidosis, tumor lysis syndrome, and exposure to nephrotoxic medications such as bisphosphonates 3, 4. Deterioration in renal function or renal failure may complicate clinical management of patients with multiple myeloma, requiring dose modification or delay of anti‐myeloma therapy, including stem cell transplant 5, 6.

The prevalence of renal impairment, chronic kidney disease, and renal failure in the multiple myeloma population varies depending on the definition and stage of disease, but has been reported to affect 20%–50% of patients with multiple myeloma, either at the time of diagnosis or emerging during the course of the disease 7, 8, 9, 10. Insufficient kidney function in multiple myeloma is associated with poor prognosis and increased mortality 7, 11, 12, 13. Adding to the kidney damage caused by their disease, patients with multiple myeloma may receive treatments that are nephrotoxic, such as chemotherapy, targeted anti‐cancer agents, and supportive care such as analgesics, antibiotics, and intravenous (IV) bisphosphonates.

This retrospective study using the Oncology Services Comprehensive Electronic Records (OSCER) electronic medical records database was designed to estimate the prevalence of renal impairment and chronic kidney disease in a current population of patients with multiple myeloma, and to describe the use of nephrotoxic agents (core anti‐myeloma therapies: doxorubicin, cisplatin, and epirubicin; and supportive care with metoclopramide or intravenous bisphosphonates including zoledronic acid or pamidronate) in these patients.

## Materials and Methods

### Study design and patients

This was a retrospective observational cohort study using the OSCER database 11 which includes electronic medical records for over 750,000 patients from over 200 outpatient practice groups across the US from 2004 to present day. Eligible patients in the OSCER database had a first diagnosis of multiple myeloma (ICD‐9CM: 203.0x), defined as the index date, between January 1, 2012 and March 31, 2015. Patients were ≥18 years of age at the index date. Exclusion criteria included a history of multiple myeloma during the 6 months prior to the index date (baseline period), solid tumor diagnosis (neoplasm) (ICD9‐CM: 140.xx‐165.xx, 170.xx‐176.xx, 179.xx‐208.xx, 210.xx, 239.xx), end‐stage renal disease and/or dialysis at baseline. Patients' demographic and clinical characteristics were collected for the 6‐month baseline period prior to the index date. The follow‐up period for each patient started at the first diagnosis of multiple myeloma (the index date) and continued until death (if documented), the last record of any kind captured for the patient, or June 30, 2015.

### Endpoints

For the primary analysis of the prevalence of renal impairment, patients were required to have ≥1 serum creatinine value recorded on or after the index date. Serum creatinine values were used to calculate estimated glomerular filtration rate (eGFR) using the Chronic Kidney Disease Epidemiology Collaboration equation). Renal impairment was defined as a single occurrence of eGFR <60 mL/min per 1.73 m^2^ on or after the diagnosis of multiple myeloma. The time to renal impairment was determined by the number of months from the diagnosis of multiple myeloma to the qualifying eGFR value.

Chronic kidney disease was defined as the presence of at least two eGFR values <60 mL/min per 1.73 m^2^ after the diagnosis of multiple myeloma that had been measured at least 90 days apart 12. The prevalence of chronic kidney disease was calculated as the proportion of patients with the event at any time among all patients who had fulfilled the testing criteria as defined in the previous sentence. Chronic kidney disease prevalence was also described in patients with renal impairment. The time to chronic kidney disease was determined by the number of months from the diagnosis of multiple myeloma to the first as well as to the confirming (second) test.

The prevalence of renal impairment and chronic kidney disease were also described in the first 6 months and first 12 months after the diagnosis of multiple myeloma. These analyses were evaluated based on patients with the requisite serum creatinine values available, qualifying them for the event within the time period and over the full study follow‐up period. Sensitivity analyses included the prevalence and time to event of renal impairment or chronic kidney disease in all patients, regardless of the availability of serum creatinine values. Time to renal impairment was also calculated for the subset of patients who did not have renal impairment at baseline.

The use of nephrotoxic agents including core anti‐myeloma therapies (doxorubicin, cisplatin, and epirubicin) and supportive care (metoclopramide; intravenous bisphosphonates, zoledronic acid or pamidronate) was described before and after either the first lowest post diagnosis eGFR value (in the case of renal impairment) or the confirming eGFR value (in the case of chronic kidney disease). Usage of these nephrotoxic agents was calculated before and after the lowest eGFR during the study period by the eGFR category (<15, 15–29, and 30–59 mL/min per 1.73 m^2^)^13^. For sensitivity analyses among patients with renal impairment, use of nephrotoxic agents before and after the lowest eGFR was assessed in the subgroup of patients that did not have improvement in renal function after the lowest eGFR during the follow‐up period of up to 12 months.

### Statistical methods

All analyses were descriptive. Kaplan–Meier methodology was used to assess the time to renal impairment and chronic kidney disease from the diagnosis date of multiple myeloma. As a sensitivity analysis, the Kaplan–Meier analysis was conducted excluding the patients with baseline renal impairment.

## Results

### Patients

**Figure 1:**
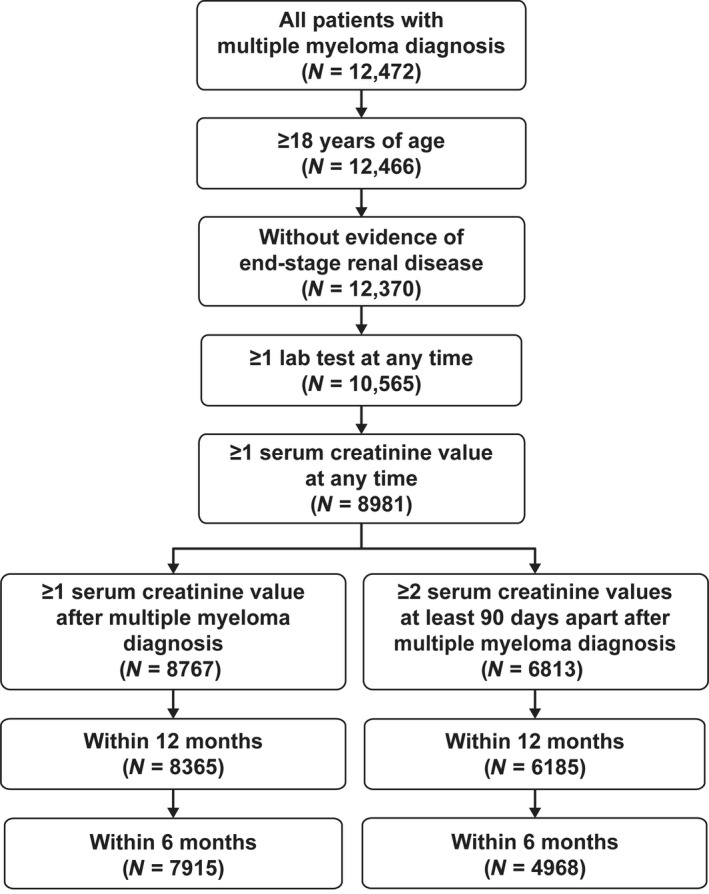
Patient selection from the Oncology Services Comprehensive Electronic Records (OSCER) database.

The OSCER database contained 12,472 patients with a diagnosis of multiple myeloma of which 12,370 met the eligibility criteria (Fig. 1 **)**. Of the eligible patients, 8767 had a serum creatinine value recorded after the diagnosis of multiple myeloma and 6813 had two serum creatinine values recorded after diagnosis that were at least 90 days apart. An additional 745 patients had an eGFR value recorded in the database, but were lacking a serum creatinine value; these patients were not included in the analyses because the method used to calculate eGFR was unclear. During the baseline period (the 6‐month period before the diagnosis of multiple myeloma), slightly more than half (54%) of the population with at least one post diagnosis serum creatinine value were men, 62% were Caucasian and 13% were African American, and the mean (SD) age was 69 11 years (Table 1). Patients with unknown race/ethnicity (12%) were assumed to be non‐African American for their individual eGFR calculation. This assumption was based on the observed overall racial distribution of the study population (13% African American and 75% non‐African American). All geographic regions of the United States were represented in the sample, although patients were concentrated in the West North Central region (Iowa, Kansas, Minnesota, Missouri, North Dakota, Nebraska, South Dakota; 39% of patients), reflecting the distribution of OSCER sites across the nation. In the subset of patients with an available baseline serum creatinine value (*N* = 2835), the mean (SD) baseline eGFR was 65 (24) mL/min per 1.73 m^2^. The median (range) number of eGFR values calculated was 8 (1, 43) during the first 12 months of follow up and 10 (1, 79) at any time during the follow‐up period. The median (range) total follow‐up time for the study was 14.3 (0.0, 43.0) months after the diagnosis of multiple myeloma.

**Table 1:** Baseline demographic and clinical characteristics

	All patients (*N* = 8767)
Age at diagnosis of multiple myeloma, years	
Mean (SD)	69 (10, 79)
Median (Q1, Q3)	70 (62, 78)
Male, *N* (%)	4750 (54%)
Race/ethnicity, *N* (%)	
African American	1153 (13%)
Asian	137 (2%)
Caucasian	5432 (62%)
Hispanic	3 (0%)
Other	974 (11%)
Unknown	1068 (12%)
Region of United States, *N* (%)	
East North Central	1023 (12%)
East South Central	479 (6%)
Middle Atlantic & New England	1637 (19%)
Mountain	343 (4%)
Pacific	158 (2%)
South Atlantic	954 (11%)
West North Central	3434 (39%)
West South Central	226 (3%)
Baseline^a^ eGFR, mL/min per 1.73 m^2^	
Patients with an available serum creatinine value, N	2835
Mean (SD)	65 (24.2)
Category, *N* (%)	
≥60	1660 (18.9%)
≥30–59	938 (10.7%)
≥15–29	237 (2.7%)
<15	excluded

The US census regions listed are comprised of the following states: East North Central (IL, IN, MI, OH, WI), East South Central (AL, KY, MS, TN), Middle Atlantic and New England (CT, MA, ME, NH, NJ, NY, PA, RI, VT), Mountain (AZ, CO, ID, MT, NM, NV, UT, WY), Pacific (AK, CA, HI, OR, WA), South Atlantic (DC, DE, FL, GA, MD, NC, SC, VA, WV). West North Central (IA, KS, MN, MO, ND, NE, SD), West South Central (AR, LA, OK, TX).

a Before the diagnosis of multiple myeloma.

### Prevalence of renal impairment

**Figure 2:**
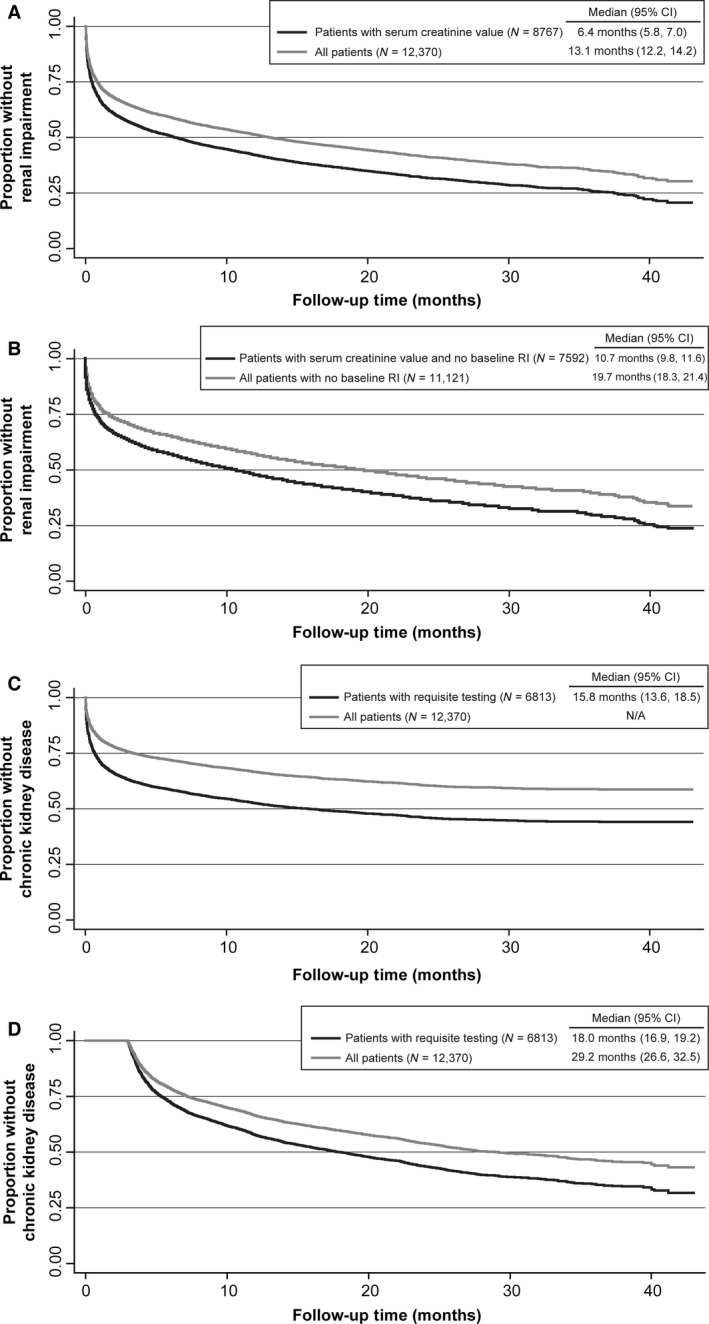
Kaplan–Meier estimated time to event analyses. (A) Time from diagnosis of multiple myeloma to renal impairment (including those with renal impairment at baseline). (B) Time from diagnosis of multiple myeloma to renal impairment in patients who did not have renal impairment at baseline. (C) Time from diagnosis of multiple myeloma to first low eGFR value in patients ultimately confirmed with chronic kidney disease. (D) Time from diagnosis of multiple myeloma to confirming eGFR value for chronic kidney disease in patients. eGFR, estimated glomerular filtration rate using the Chronic Kidney Disease Epidemiology Collaboration equation.

A total of 8767 patients had at least one serum creatinine value recorded after the diagnosis of multiple myeloma and were included in the primary analysis of the prevalence of renal impairment. Of these, 5334 patients or 61% (95% CI: 60, 62) experienced renal impairment during their follow‐up period (Table 2). The median (95% CI) time from diagnosis of multiple myeloma to renal impairment was 6.4 (5.8, 7.0) months (Fig. 2A). In patients with an available serum creatinine value and no renal impairment at baseline (*N* = 7592), the median (95% CI) time to renal impairment was 10.7 (9.8, 11.6) months (Fig. 2B).

**Table 2:** Prevalence of renal impairment and chronic kidney disease after diagnosis of multiple myeloma

	Cases/population	Prevalence percentage (95% CI)
Renal impairment		
Over follow‐up period, primary analysis	5334/8767	61 (60, 62)
6 months^a^	4159/8767	47 (46, 48)
Among patients with serum creatinine value	4159/7915	53 (51, 54)
12 months^a^	4725/8767	54 (53, 55)
Among patients with serum creatinine value	4725/8365	56 (55, 58)
Chronic kidney disease		
Over follow‐up period, primary analysis	3399/6813	50 (49, 51)
6 months^a^	1830/6813	27 (26, 28)
Among patients with requisite testing^b^	1830/4968	37 (35, 38)
12 months^a^	2676/6813	39 (38, 40)
Among patients with requisite testing^b^	2676/6185	43 (42, 45)

a After diagnosis of multiple myeloma.

b Two eGFR values <60 mL/min per 1.73 m^2^ at least 90 days apart.

Forty‐seven percent (*N* = 4159 of 8767) of those with at least one serum creatinine measurement at any time during the study experienced renal impairment within 6 months of the multiple myeloma diagnosis and 54% (*N* = 4725) experienced renal impairment within twelve months of the diagnosis. For patients with at least one serum creatinine value recorded during the respective time period (i.e., those in whom renal impairment could have been detected), the prevalence of renal impairment was 53% (*N* = 4159 of 7915) within 6 months of diagnosis and 57% (*N* = 4725 of 8365) within 12 months of diagnosis.

Among all 12,370 patients who met the eligibility criteria (diagnosis of multiple myeloma, at least 18 years of age, and did not have end‐stage renal disease), regardless of the availability of serum creatinine values, the prevalence of renal impairment was 43% (95% CI: 42, 44) detected a median 13.1 months (95% CI: 12.2, 14.2) after the multiple myeloma diagnosis (Fig. 2A). Considering only those who did not have renal impairment at baseline (*N* = 11,121), the median (95% CI) time to renal impairment was 19.7 (18.3, 21.4) months (Fig. 2B).

### Prevalence of chronic kidney disease

A total of 6813 patients had at least two serum creatinine values recorded at least 90 days apart after the diagnosis of multiple myeloma and could be evaluated for the primary analysis of the prevalence of chronic kidney disease. Fifty percent (*N* = 3399; 95% CI: 49, 51) of these patients experienced chronic kidney disease during their follow up (**Table** 2). Twenty‐seven percent (*N* = 1830) had detectable chronic kidney disease within 6 months and 39% (*N* = 2676) had detectable chronic kidney disease within 12 months after the diagnosis of multiple myeloma. For patients with at least two available serum creatinine values at least 90 days apart during the requisite period, the prevalence of chronic kidney disease was 37% within 6 months and 43% within 12 months after diagnosis of multiple myeloma. The prevalence of chronic kidney disease over the entire follow‐up period (unique for each patient) in all eligible patients (*N* = 12,370) regardless of the availability of serum creatinine values was 28% (sensitivity analysis). Among the 5334 patients with renal impairment, 3399 (80%) had evidence of chronic kidney disease over the course of follow up.

The median (95% CI) estimated time to chronic kidney disease after the diagnosis of multiple myeloma was 15.8 (13.6, 18.5) months to the first of the two required eGFR values (Fig. 2C) and 18.0 (16.9, 19.2) months to the second (confirmatory) eGFR value (Fig. 2D). Note that the initial flat line of the first curve reflects the 90‐day period in which no events can occur by definition.

### Usage of nephrotoxic medications (doxorubicin, metoclopramide, cisplatin, epirubicin, intravenous bisphosphonates)

In the 5334 patients with renal impairment, the use of nephrotoxic agents or specifically bisphosphonates did not notably change from before the lowest eGFR value (median time from diagnosis of multiple myeloma, 8.3 months) to 12 months after the lowest eGFR value (median follow‐up time, 10.5 months)(Table**** 3). However, in patients with severe renal impairment (eGFR <15 mL/min per 1.73 m^2^), reduced use of these agents was observed after the lowest eGFR (nephrotoxic agents from 27% before to 23% after; bisphosphonates from 23% before to 15% after). Decreases after the lowest eGFR were also observed in the group with eGFR 15–29 mL/min per 1.73 m^2^ (nephrotoxic agents from 38% to 34%; bisphosphonates from 35% to 30%) (Table 3). In the subgroup of patients with renal impairment who did not experience chronic kidney disease stage improvement after the lowest eGFR value (*N* = 1463), the use of any nephrotoxic agent prior to the lowest eGFR was reduced within the 12 months after the lowest eGFR (38% vs. 29%, respectively, for all renal function categories); however, in the lowest renal function categories, the reduction of nephrotoxic agents use was halved (41% vs. 20%, respectively, in 264 patients with eGFR 15–29 mL/min per 1.73 m^2^, and 18% vs. 9%, respectively, in 170 patients with eGFR <15 mL/min per 1.73 m^2^). Similarly, in this same subgroup, use of IV bisphosphonates before and after the lowest eGFR value was 38% and 29%, respectively, overall, while use was halved in the lower renal function categories (41% vs. 20% in those with eGFR 15–29 mL/min per 1.73 m^2^, and 17% vs. 9%, respectively, in those with eGFR <15 mL/min per 1.73 m^2^).

**Table 3:** Use of nephrotoxic agents in patients with renal impairment or chronic kidney disease by eGFR category

	Lowest eGFR (renal impairment) or confirming eGFR (chronic kidney disease) category (mL/min per 1.73 m^2^) in study period			
	<60	30–59	15–29	<15
Patients with renal impairment^a^ (*n*, %)	5334 (61%)	3488 (40%)	1220 (14%)	626 (7%)
Any nephrotoxic agent before lowest eGFR value	2109 (40%)	1472 (42%)	465 (38%)	172 (27%)
Any nephrotoxic agent within 12 mo of lowest eGFR^b^	2057 (39%)	1497 (43%)	413 (34%)	147 (23%)
Any IV BP before confirming eGFR	1981 (37%)	1404 (40%)	430 (35%)	147 (23%)
Any IV BP within 12 mo of confirming eGFR^b^	1862 (35%)	1402 (40%)	365 (30%)	95 (15%)
IV BP plus other nephrotoxic agent within 12 mo	205 (4%)	129 (4%)	55 (5%)	21 (3%)
Time to lowest eGFR (months), median (Q1, Q3)	2.3 (0.4, 11.5)	3.4 (0.7, 12.9)	1.2 (0.2, 7.3)	1.0 (0.2, 6.0)
Time after lowest eGFR (months, maximum 360), median (Q1, Q3)	10.5 (3.8, 11.8)	9.9 (3.4, 11.8)	11.8 (5.2, 11.8)	8.8 (3.7, 11.8)
Patients with chronic kidney disease^c^ (*n*, %)	3399 (50%)	2006 (29%)	944 (14%)	449 (7%)
Any nephrotoxic agent before confirming eGFR	1537 (45%)	999 (50%)	407 (43%)	131 (29%)
Any nephrotoxic agent within 12 mo of confirming eGFR^b^	1567 (46%)	962 (48%)	455 (48%)	150 (33%)
Any IV BP before confirming eGFR	1466 (43%)	967 (48%)	386 (41%)	113 (25%)
Any IV BP within 12 mo of confirming eGFR^b^	1441 (42%)	911 (45%)	423 (45%)	107 (24%)
Use of IV BP plus other nephrotoxic agent within 12 mo	186 (5%)	87 (4%)	70 (7%)	29 (6%)
Time to lowest eGFR (mo), median (Q1, Q3)	7.7 (4.4, 16.9)	10.0 (5.3, 19.4)	5.6 (3.9, 12.6)	4.9 (3.6, 11.5)
Time after lowest eGFR (mo, maximum 360), median (Q1, Q3)	10.0 (3.6, 11.8)	9.3 (3.2, 11.8)	11.8 (5.2, 11.8)	8.3 (3.0, 11.8)

eGFR, estimated glomerular filtration rate; IV BP, intravenous bisphosphonate.

a In the total patient sample (*N* = 8767).

b Patients not required to have 12 complete months of follow up.

c Percentage among all patients with at least two eGFR values at least 90 days apart (*N* = 6813).

Among the 3399 patients with chronic kidney disease, 1537 (45%) received any nephrotoxic agent before the confirming eGFR, while 1567 (46%) received it within 12 months after the confirming eGFR (median follow‐up time, 10.0 months). Similarly, 1466 (43%) patients received an IV bisphosphonate prior to their confirming eGFR value, while 1441 (42%) received them within the 12 months after the confirming eGFR. In contrast to the patients with renal impairment, the use of either any nephrotoxic agent or bisphosphonate remained similar before and after the confirming eGFR value in all eGFR categories (30–59, 15–29, <15 mL/min per 1.73 m^2^) in those with chronic kidney disease.

## Discussion

This retrospective study of 12,370 patients with multiple myeloma showed a high prevalence of both renal impairment and chronic kidney disease (61% and 50%, respectively) in patients treated in oncology clinics in the US between 2013 and 2015 (OSCER cancer database). Furthermore, the onset of both conditions was rapid, with a 6‐month prevalence of 47% for renal impairment and 27% for chronic kidney disease after the multiple myeloma diagnosis. These results, which were observed in a recent population with newly diagnosed multiple myeloma using the current standard definitions for renal impairment and chronic kidney disease, are comparable to or higher than the prevalence reported in older datasets dating from the 1970s, 1980s, and 1990s 7, 8, 9, 10.

We observed that onset of renal impairment occurred within a median 6 months following the diagnosis of multiple myeloma while the onset of chronic kidney disease occurred within a median 18 months, with 80% of those with renal impairment subsequently showing evidence of chronic kidney disease. In patients who did not have renal impairment at baseline, the onset of renal impairment was slightly slower (median 10.7 months). We therefore conclude that renal impairment is prevalent with a rapid onset in those with newly diagnosed multiple myeloma, and the majority of those with renal impairment progress to chronic kidney disease.

Despite the presence of renal impairment, a substantial proportion of patients nevertheless received nephrotoxic agents (predominantly bisphosphonates). Anticipation or realization of some measure of renal function improvement after myeloma treatment may explain the continued use of nephrotoxic agents after the lowest eGFR (i.e., the worst renal function experienced by the patients during their observation period); however, despite treatment, almost 20% of patients still do not show improvement in renal function 14. This lack of renal recovery is believed to be multifactorial, including lack of response to treatment. We observed that only patients in the two most severe renal impairment categories (eGFR <15 mL/min and 15–29 mL/min per 1.73 m^2^) had reduced use of nephrotoxic agents and bisphosphonates after the lowest eGFR. These results highlight the unmet need in patients with multiple myeloma, who, despite the presence of renal impairment, continued to receive bisphosphonate treatment to maintain bone health in the absence of a non‐nephrotoxic treatment choice. In the subgroup of patients who did not have improvement in renal function after the lowest eGFR, use of these agents was mostly halved, indicating that the unmet need in this population is even greater, as they could not continue use of nephrotoxic anti‐myeloma agents or intravenous bisphosphonates. Similarly in the subset of patients without chronic kidney disease stage improvement after the lowest eGFR, both nephrotoxic agent use and bisphosphonate use were halved after renal impairment in patients in the two lowest eGFR categories.

Kidney function is expected to improve with the novel multiple myeloma therapies; however, a recent study demonstrated that even when patients with newly diagnosed multiple myeloma experienced resolution of their renal impairment upon myeloma treatment, survival outcomes remained worse compared to the population without renal impairment 14. Therefore, our results suggest that more attention should be placed on the avoidance of nephrotoxic drug use in this setting, including a consideration of alternatives to bisphosphonates.

The limitations of this study include those common in observational research and retrospective databases, such as potential misclassification in diagnosis codes and laboratory results. In our dataset, socio‐demographics such as gender, race, and region were well populated, but comorbidities were not fully captured through ICD9 codes. The OSCER database records the experience of the patients treated primarily at oncology/hematology clinics, which may affect the generalizability of the results to patients treated in other settings. A minimum of one serum creatinine record after multiple myeloma diagnosis was required for inclusion in the primary analysis cohort, resulting in the exclusion of 3603 (29%) of eligible patients in the OSCER database. We are unable to determine whether these patients were not receiving renal assessment, or whether the renal assessment was occurring outside the oncology practice. Since patients with poor renal function may have received more frequent renal assessment, selection bias may have been introduced by the requirement for a serum creatinine value. To address this potential bias, we calculated renal impairment and chronic kidney disease prevalence including all 12,370 patients, and both remained high. The assumption that those with unknown race were non‐African American for the eGFR calculation was based on the racial distribution of the overall sample; however, if this assumption was incorrect, results could have been biased toward higher prevalence of renal impairment, since the eGFR of African Americans is lower than non‐African Americans. We excluded patients with evidence of end‐stage renal disease because these patients represent a unique population that is likely to receive different treatment at baseline. Although patients with end‐stage renal disease were previously reported to represent 9% of the multiple myeloma population)^8^, in the newly diagnosed myeloma population in our study, only 96 patients (0.8%) were excluded due to end‐stage renal disease; therefore, the impact on the calculation of the prevalence of renal impairment in our study would have been negligible. The time‐to‐event analyses are subject to detection bias due to the lack of patient history prior to the diagnosis of multiple myeloma; thus, patients may have fulfilled the definitions of renal impairment and/or chronic kidney disease earlier than indicated by their post diagnosis records. A final limitation of the study was the difficulty in assessing use of non‐steroidal anti‐inflammatory drugs, an additional type of nephrotoxic agent used in the myeloma population. Due to frequent over the counter use, these agents are under‐reported in the oncology practice electronic medical records and cannot be accounted for reliably.

Future studies, such as determination of the economic burden of renal impairment in multiple myeloma and the impact of renal impairment on anti‐myeloma treatment patterns, could contribute to a better understanding of the burden of renal impairment in the multiple myeloma population.

## Conclusion

The prevalence of both renal impairment and chronic kidney disease was high in patients with multiple myeloma, affecting 61% and 50%, respectively, of patients in the OSCER cancer database in the US for the period 2012 to 2015. The onset of renal impairment was rapid after the multiple myeloma diagnosis; however, 40% of these patients nevertheless received concomitant nephrotoxic agents, most of which were intravenous bisphosphonates. As renal impairment is associated with reduced survival and may affect clinical management, preservation of renal function is critical, and non‐nephrotoxic alternatives are warranted where possible in managing the multiple myeloma population.

## Conflict of Interest

Yi Qian, Debajyoti Bhowmik, Rohini K. Hernandez, Paul Cheng, and Michele Intorcia are employees of Amgen Inc. and hold Amgen stock. Steven Wang, and Sam Colman are employees of Covance, Gaithersburg Maryland, USA. Christopher Bond was an employee of Covance when the work was conducted. Covance received remuneration from Amgen for this study.
